# Atrophic Dermatofibrosarcoma Protuberans with Eosinophilic Infiltration

**DOI:** 10.3390/dermatopathology9040044

**Published:** 2022-11-27

**Authors:** Anber Mahboob, Claire Turgeon, Syeda Qasim, Arif Usmani

**Affiliations:** 1 Sharif Medical and Dental College, Lahore 55150, Pakistan; 2 University of Toledo Medical Center, Toledo, OH 43606, USA; 3 RWJBarnabas Health, Livingston, NJ 07052, USA; 4 Benchmark Diagnostics, Middleburg Heights, OH 44130, USA

**Keywords:** dermatofibrosarcoma protuberans, atrophy, eosinophilia

## Abstract

Dermatofibrosarcoma protuberans (DFSP) is a rare, locally aggressive spindle cell mesenchymal tumor arising in the dermis, with low metastatic potential. The most commonly affected sites are the trunk and proximal extremities; rarely are acral sites involved. Atrophic DFSP is a rare form of DFSP, that is morphologically different but histologically similar to DFSP. It commonly affects young adults between the ages of 20 to 50 years. The current management strategy for atrophic DFSP is surgical excision with long-term follow-up to detect any recurrence. Only one known case of atrophic DFSP with eosinophilic infiltration is what makes our case an exceptionally rare presentation.

## 1. Introduction

Dermatofibrosarcoma protuberans (DFSP) is a rare cutaneous tumor representing 1.8% of all soft tissue sarcomas [1,2,3]. The WHO classified DFSP as a low-grade sarcoma, owing to its locally aggressive behavior and low metastatic potential [2,4]. It primarily affects trunks and extremities and rarely involves other body sites [2]. This tumor has a higher propensity for women and the black population [2,5,6]. The most common clinical presentation of DFSP is a single, slow-growing, pinkish or brownish asymptomatic plaque-like area of cutaneous thickening or raised, nipple-like projection, which usually takes years to develop, causing latency in seeking medical attention, and delayed diagnosis [2,7]. The atrophic variant was first described by Lambert et al. in 1985 [8,9]. Eosinophilic infiltration of atrophic DFSP has been reported in only one case to date [10]. We report another case of atrophic DFSP with eosinophilic infiltration in a young female patient.

## 2. Material and Method

**Figure 1:**
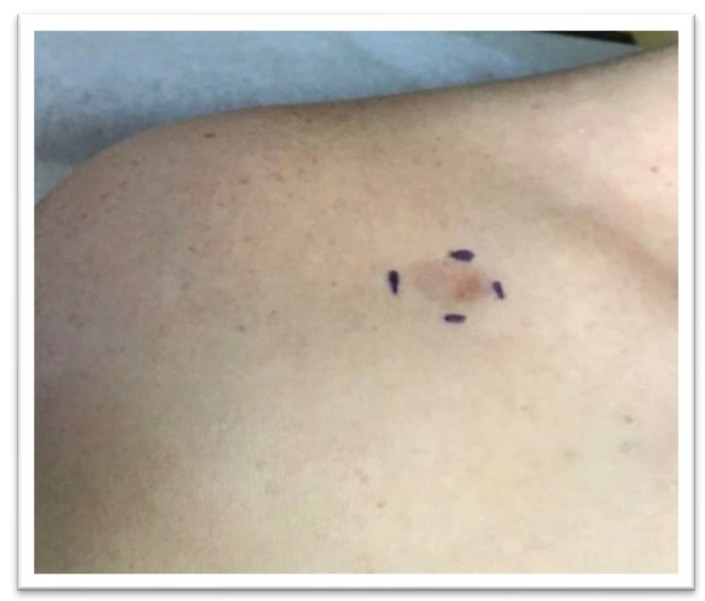
A 2.5 cm erythematous to violaceous atrophic patch with a firm papule at the inferior lateral aspect.

**Figure 2:**
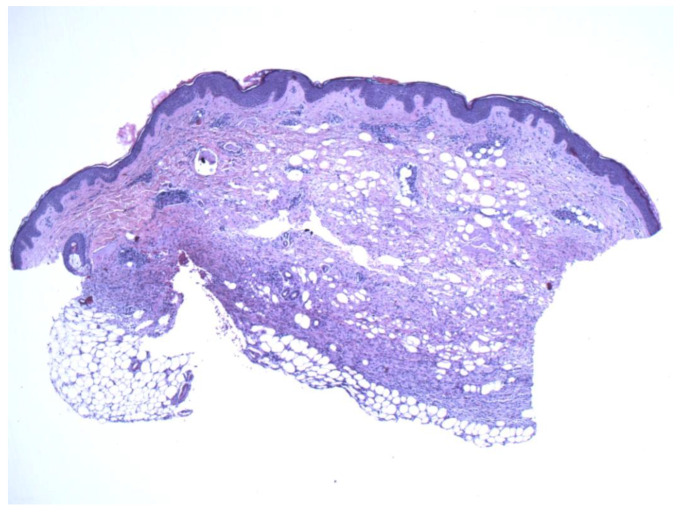
A thin strip of spindle cells is noted in the deeper dermis with subjacent fat. The mid and superficial dermis show sparse spindle cells with atrophic changes (4×).

**Figure 3:**
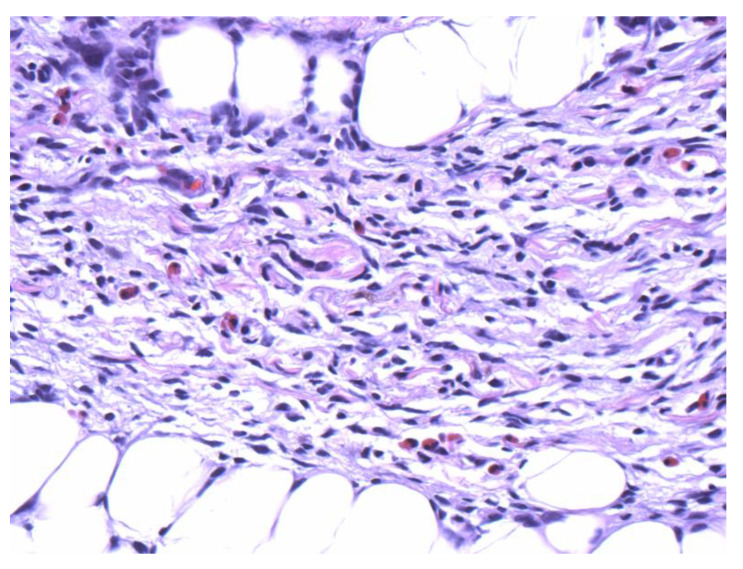
Numerous eosinophils are scattered within the spindle cell proliferation (40×).

**Figure 4:**
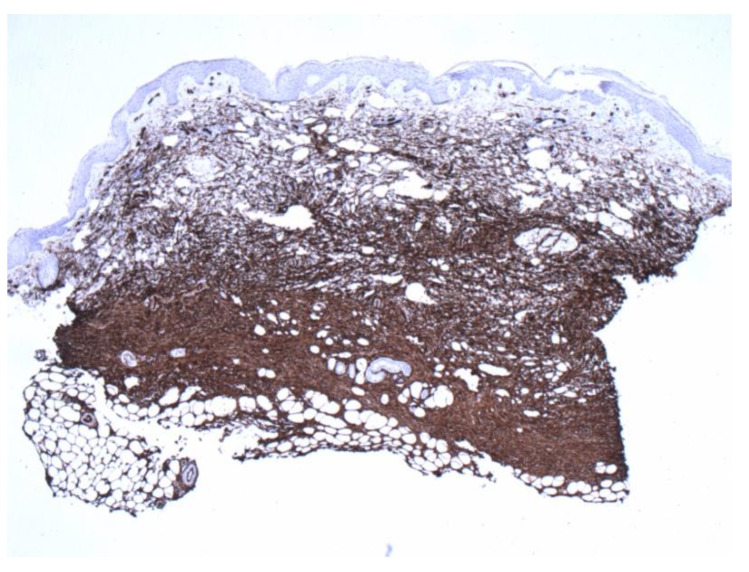
Positive CD34 staining with contrasting sparse-superficial and dense-deeper areas (4×).

**Figure 5:**
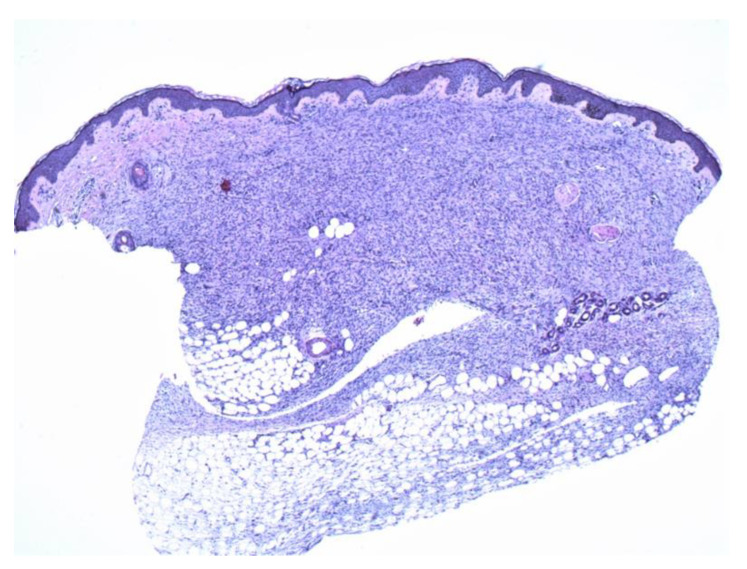
The second biopsy shows diffuse and even involvement of the dermis (4×).

**Figure 6:**
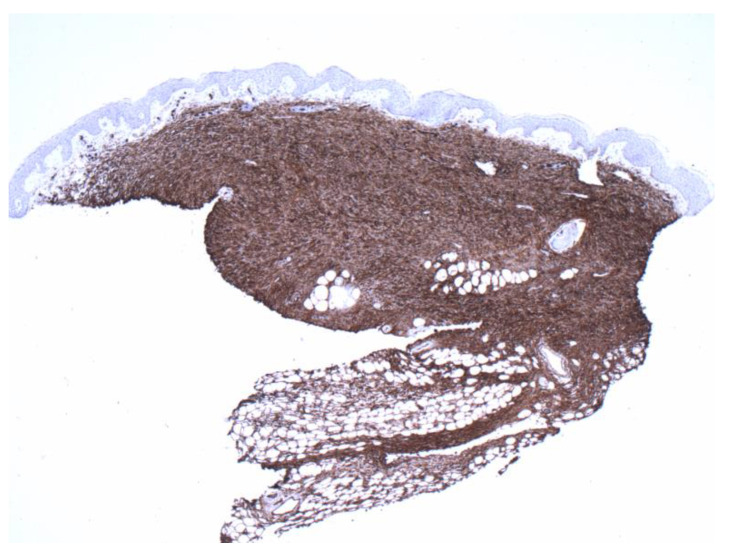
Diffuse and even staining was also highlighted by CD34 preparation, compared with Figure 4. (4×).

The patient is a 27-year-old female who presented to the dermatology office with a skin lesion on the right anterior shoulder. The lesion had been present first as an area of discoloration for 2–3 years. Several weeks before presentation, the lesion changed texture and developed a focal papular area. The patient had no pertinent past medical or surgical history and no known drug allergies. A 2.5 cm erythematous to violaceous atrophic patch with an isolated, slightly firm papule, was noted in the right anterior shoulder distribution (Figure 1). At the time of her initial visit, a 0.4 cm punch biopsy of the papular component of the lesion was performed. The histologic examination demonstrated a deep dermal strip of spindle cell proliferation also involving the subcutaneous fat (Figure 2). A higher magnification exam revealed numerous intervening eosinophils (Figure 3). The superficial dermis showed sparsely dispersed spindled cells. The spindle cells diffusely expressed CD34 antigen (Figure 4). Due to the unusual features, a second punch biopsy was performed four weeks later (Figure 5). This biopsy showed an extensive mildly atypical dermal and subcutaneous CD34+ spindle cell proliferation without significant eosinophils, compatible with a more typical histologic presentation of dermatofibrosarcoma protuberans (Figure 6). The spindle cells did not express S100, sox-10, actin, or desmin antigens with valid internal and external controls. A negative immunohistochemical study for neural, melanocytic, myofibroblastic, and muscle markers further confirmed the diagnosis.

## 3. Discussion

Various subtypes of DFSP have been described, including atrophic, granular, myxoid, sclerosing, fibrosarcomatous, and pigmented (Bednar Tumor) [1,11,12,13]. Clinically, atrophic DFSP lesion is centrally depressed, whereas classical DFSP has a protuberant presentation; however, both variants show similar histological and immunohistochemical features, such as fibrohistocytic cell proliferation [14,15], CD34, and vimentin expression, and lack of staining with CD44, S100 protein or XIIIa [1,16]. Both variants also demonstrate COL1A1/PDGFB gene rearrangement fusion [2,16].

Tissue eosinophilia in this lesion is a highly unusual finding. Eosinophilia is a frequently encountered finding in malignant neoplasms that is considered a favorable host response to malignant tissue [10]. Eosinophils have an anti-tumorigenic (e.g., TNF-α, granzyme, cationic proteins, and IL-18) and protumorigenic role in different cancers, depending on the location. In melanoma and some gastrointestinal cancers, eosinophils play an anti-tumorigenic role, while in Hodgkin’s lymphoma and cervical carcinoma have been linked to poor prognosis. In melanoma, tumor-associated eosinophils express chemokines (CCL5, CXCL9, CXCL10), prompting the recruitment of tumor-reactive CD8 T cells that mediate tumor rejection. Moreover, eosinophils may collaborate with macrophages, mast cells, NK cells, and dendritic cells to induce anti-tumor responses. Eosinophils may also affect local T cell responses, by modulating the balance of Th1 and Th2-related cytokines. Lastly, eosinophils may also serve as antigen-presenting cells (APC).

Atrophic DFSP can be confused with morphea, atrophic dermatofibroma, morpheaform basal cell carcinoma, resolving panniculitis, and lipoatrophy [1,16]. The coexistence of spindle cell proliferation and eosinophils may be seen in disparate conditions like dermatofibroma with eosinophils, late-stage erythema elevatum diutinum (EED), fibrosis caused by ruptured cysts, and resolving insect bite reaction. However, dermatofibroma is typically CD34 negative. Fibrosis in late-stage EED can be storiform, but the presence of neutrophils and plasma cells is not seen in our case. Finally, fibrosis caused by the ruptured cyst and insect bite reaction is not storiform and is usually associated with a granulomatous reaction.

Surgical excision remains the mainstay of treatment [14]. Histological margins are usually more extensive than clinical margins; hence wide local excision is generally considered a preferred treatment modality with a safety margin range from 3 to 5 cm, especially if the lesion is not in delicate areas such as the head and neck, where Mohs Micrographic Surgery (MMS) is preferred to avoid cosmetic side effects. Re-excision should be performed in case of recurrence [2]. Distant metastasis has been reported in 6% of patients despite multiple failed local surgery attempts [2,17].

In our case, these changes were thought to be consistent with a diagnosis of dermatofibrosarcoma protuberans with eosinophilic infiltration. However, the differential diagnosis included a fibrotic reaction to an insect bite, or an unusual inflammatory response to a possible cyst or traumatic event. Granulomatous diseases were not considered in our differential diagnoses, as no granulomatous or histiocytic component was noted in either of the biopsy specimens. Demonstration of diffuse expression of CD34, in the initial and subsequent biopsy and characteristic histopathology in the subsequent biopsy, confirmed the diagnosis of DFSP.

## 4. Conclusions

We report a rare case of atrophic DFSP with eosinophilic infiltration. DFSP should be considered in the differential diagnosis, while evaluating an atrophic cutaneous plaque to avoid delays in diagnosis. An evidenced-based defensive immunological role of eosinophils has been postulated in certain malignancies [18]. However, further studies are required to understand the exact correlation between eosinophilia and atrophic DFSP and its impact on prognosis. The findings here may be related to an unusual immunological response to DFSP, or merely reflect an arthropod encounter.

## Data Availability

Data supporting reported results can be found on PubMed and Google Scholar.
